# Connecting links with code: AI in morphological evolution

**DOI:** 10.3389/fbinf.2026.1825851

**Published:** 2026-06-22

**Authors:** Nimisha Singh, Pragya Chaturvedi, Devansh Saxena

**Affiliations:** Department of Biotechnology, Dr. B. Lal Institute of Biotechnology, Jaipur, Rajasthan, India

**Keywords:** artificial intelligence, deep learning, morphology, morphometric analysis, phylogenetics

## Abstract

Evolutionary biology and paleontology are based on fossil material of morphological data, i.e., anatomical, size, symmetry, ontogenetic, etc., as the dominant empirical record of the history of life over deep time. Such physical records tie evolutionary histories to the geologic past, guiding evolutionary molecular clock calibrations, phylogenetic speculations and macroevolutionary trends such as adaptive radiations, mass extinctions, morphological stasis and disparity. Additional explanations of the history of biogeography, evolution inside niches and lineage adaptation to environmental disturbances are provided by spatial-temporal variability in the fossil record. Although central, traditional morphological studies are characterized by methodological shortcomings: manual scoring of characters, linear measurements and landmark-based geometric morphometrics which causes subjectivity, homoplasy and absence of data and topological instability in phylogenies. The present review features fields in evolutionary biology and paleontology that can be improved by AI and machine learning developments in processing morphological data, which quantitative shape analysis automates, reduces biases and scales inference, which can prove. We have discovered technical and methodological gaps and have mapped out ways of integrating these tools into strong, repeatable workflows.

## 1 Introduction

Evolutionary biology and paleontology are concerned with the study of the form of organisms and their evolution over deep time. The intricate anatomical, morphological, size, symmetry and ontogenetic data offer the major empirical material to be used to retrieve the history of life on Earth. Fossils are the unique tangible records of dead organisms; they serve as valuable media through which evolutionary histories ground themselves in geological time. They are not just useful as a dead asset, but active sources of information, which can be important in measuring molecular clocks, in evaluating competing phylogenetic theories and in understanding macroevolutionary phenomena such as adaptive radiations, mass extinctions and times of morphological stasis or disparity (Yu et al., 2024).

The spatial and temporal distribution of morphological variability in the fossil record can enable scientists to know about historical biogeography and to trace the evolution of ecological niches and how fossil lineages have reacted to the gross environmental disturbances over millions of years (Lamsdell et al., 2025).

Although of fundamental significance, the standard analysis of morphological and fossil data has been constantly afflicted by methodological issues that have restricted the breadth and quality of the evolutionary inference. The conventional methods have been very dependent on hand observation, manual qualitative scoring of discrete characters (e.g., presence/absence, state coding) and linear measurements with calipers or microscopes (He et al., 2024). While geometric morphometrics (GM) represented a significant advance by enabling the quantitative analysis of shape through landmark-based Procrustes superimposition, it too is hampered by practical bottlenecks (Hedrick, 2023). Phylogenetic analyses based on morphology frequently suffer from common issues of character ambiguity, extensive missing data, high levels of homoplasy (convergent evolution) and the subjective weighting of characters, all of which can lead to phylogenetic trees with low resolution, poor statistical inference, or topological instability (Wright and Hopkins, 2025).

The current review highlights the major disciplines of evolutionary biology and paleontology that have the most to gain from the recent computational development in the morphological data analysis, especially that with artificial intelligence (AI) and machine learning (ML) to break long-standing methodological constraints. The review also mentions the technical and methodological gaps that surround the morphological and fossil data analysis.

## 2 Fossil and morphological data

### 2.1 Fossil data

Prepared fossils–those ready to be exploited by researchers, or are often referred to as Fossil Data that are available for display in the museum (Watkins, 2024). According to Bokulich and Parker, “fossil rocks can be called a physical data model. In this context, the fossils are taken as a depiction of past existence on Earth” (Bokulich and Parker, 2021). This fossil data provides evidence for the past evolutionary and ecological processes, which concludes the study of palaeo-chronologies (Rodríguez-Rey et al., 2016). Fossils and geo-historical data attracted high research attention in the 1980s, which helped in tracking the trends of the history of life (Jablonski and Shubin, 2015). Fossil records are the principal source that provides the details on how the biodiversity has changed over the period of time, which reveals the long-term vitality of diversification and its factors (Benson et al., 2021). Fossil data and records are available in multiple formats for various analysis purposes and result outcomes.

Body fossils represent the hard parts of any organism, such as arthropod carapaces, mollusk shells, or bones. The Ediacaran–Cambrian period encloses unique styles of fossil preservation that also hold the soft part like eyes, traces of the gut and other projecting parts, which provide a rich insight into the evolutionary and developmental dynamics of variations (Erwin, 2020). Body fossils attracted the most attention, however other formats of fossil records did provide a solid foundation for biological history. These fossils represent numerous anatomical parts and exhibit morphological disparity that follows a Gaussian distribution, or they may display distinct variation along certain linear features, particularly when the fossils consist of bodily fragments (Tacker et al., 2010).

Trace fossils are a key indicator that helps in the evaluation of the period of diversification and ecosystem development that appeared in the course of the Cambrian explosion (Mángano and Buatois, 2014). These fossil types represent distinctive behavioral design exclusive of marine organisms. Yet certain characteristic marine ichnogenera (for example, Paleodictyon, Nereites, Scolicia, Chondrites) have been reported occasionally in continental deposits (Buatois and Mángano, 2011). Trace fossils support the thought that body fossils carried a fairly authentic record of the predominant history of large-sized metazoans. Trace fossils exhibit some distinction from the body fossils. Trace fossils are created by complete organisms and display consistent widths throughout their length and also possess uniform lengths when formed by the repeated feeding activities of a tracemaker of the same size. Genuine trace fossils should also feature levees and grooves, which suggest the organism actively shifted sediment. These fossils can also show active backfilling or contain testaments of material passed through the digestive organs, such as the fecal pellets (Tacker et al., 2010).

Ancient biomolecules such as DNA are known to provide the third line of evidence about the early organisms and are considered the most appreciated chemical fossil (Briggs and Summons, 2014). Duda and colleagues studied the presence of molecular fossils, hopanes and the absence of steranes that indicated the domination of anaerobic bacteria while the eukaryotes were ecologically minor in the Mesoproterozoic ecosystem, respectively (Duda et al., 2021).

### 2.2 Morphological data

**FIGURE 1:**
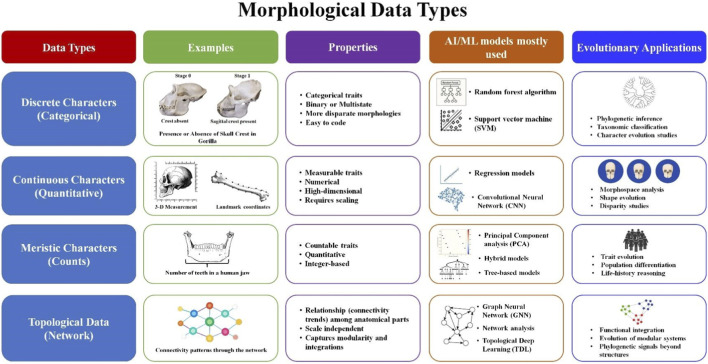
Types of morphological data commonly utilized in evolutionary analyses.

Molecular and morphological data are a necessary source of data for the comprehension of the fossil insights. Contemporary studies usually use the morphological data with molecular partitions in combined analyses (Keating et al., 2023). Morphological data have been a reliable source of primary reference for defining the vast majority of organisms and understanding their phylogenetic history, but are necessary for defining, recognizing and diagnosing pathological conditions in animals, plants and other living species (Vogt, 2019). Morphological analysis is a widely used and economical method for the taxonomic recognition of wildlife history and remains. Though used as a wide source of information in identifying species, morphological analysis is still underutilized in wildlife biology associated with DNA analysis (Trail, 2021). The morphological data can be categorized into several distinct types (Figure 1).

In macroevolutionary studies, the evaluation of morphological disparity is based on morphospaces generated from discrete character data. This shift is an outcome of the use of phylogenetic data matrices as character spaces for conducting disparity investigations (Gerber, 2019). For instance, an analysis of more than 40 coelurosaurian taxon datasets used discrete characters, such as mandibular and dentary tooth characters, that showed remarkable correlations and largely similar patterns of morphological variation (Schaeffer et al., 2020). Another study included the captorhinid taxa of 75 original characters from which 31 morphometric characters were included in the matrix after construction; 18 were of postcranial (appendicular) and 13 were of cranial morphology (Romano et al., 2017). Phylogenetic trees are generally generated from genetic data, such as aligned nucleic acids or protein sequences, gene order on chromosomes, genetic markers such as SINEs (Short Interspersed Nuclear Elements), SNPs (Single Nucleotide Polymorphisms), etc., and the gene presence/absence across taxa (Bordewich et al., 2017). The morphological characterization of taxa is described by the phylogenetic matrices as combinations of character states and thus becomes, mathematically if not conceptually, comparable to discrete datasets featured in the numerical taxonomy or constructed for disparity purposes (Hetherington et al., 2015). The fundamentals of primary and secondary homology that were originally developed for the discrete character data can be easily applied to geometric morphometric data. In this context, raw resemblance in aligned landmark coordinates accounts for primary homology, while similarity from the resultant ancestry constitutes secondary homology (Palci and Lee, 2019). Discrete characters with the phylogenetic matrices contain combinatorial states of traits, whereas continuous geometric morphometrics approaches evaluate geometric patterns of shapes. Both the techniques shed light on disparity distributions in taxa such as captorhinids and/or theropods, frequently producing identical outcomes despite poor inter-trait correlations (Schaeffer et al., 2020; Romano et al., 2017).

In contrast, geometric morphometrics (GM) emphasizes the geometry of the structures by retaining the spatial relationships among points or outlines, which allows the shape differences that can be directly visualized as deformities instead of being expressed as simple distances or ratios (Tatsuta et al., 2018). The morphological disparity data summarizes the highly multivariate morphological differences across groups of organisms within clades, time intervals, or other classes. The variation in morphology can be evaluated using geometric morphometrics, figure, or surface-based techniques (Van den Ende et al., 2023). A geometric morphometric analysis of Eriocheir sinensis (Chinese mitten crabs) used 35 standardized specimen carapace landmark points to generate mean shapes across three geographic origins, which demonstrated significant spatial morphological variation and nearly 100% classification precision (Zhang et al., 2025). The 2D or 3D coordinates of those structures return their relative locations, with variations in such areas across the objects measuring the degree of shape variation (Finka et al., 2019). For instance, D’Angelo Del Campo and colleagues compared human skull size and shape between cold-adapted and temperate zone human populations using landmark-based analyses, which demonstrated craniofacial features associated with colder regions, such as increased prognathism, larger nasal cavities and orbits, zygomatic expansion and elongated and flattened cranium were significantly observed despite the genetic dissimilarities (D’Angelo Del Campo et al., 2025). Computerized geometric morphometric techniques enable high reliability, replicability, precision and statistically significant data analysis, testing and visualization of form differences for quantitative shape investigations (Fox et al., 2020).

Since landmark location and shape variables are evaluated along a continuous measurable scale, geometric morphometrics (GM) relies mostly on the analysis of continuous characters of specimen morphology. In morphometrics, the term shape is used to represent the geometric features of any specimen that are independent of the orientation, size and location, while the specimen configuration comprises both its size and shape (Mitteroecker and Gunz, 2009; Slice, 2007). The continuous characters, also called quantitative characters, are ratios of measurements or dimensions of purportedly homologous components. The traits of continuous characters, such as skull shape scores, body length, or centroid size, are measured on a numerical scale that can have any value within a range (Palci and Lee, 2019; Klingenberg, 2016). Characters such as cranial breadth, body length, or shape scores differ continuously within the ranges, preventing the information loss linked to discrete coding. They outperform the Mk models under Brownian motion and enable objective measurement free from researcher subjectivity, in contrast to discrete coding (Parins-Fukuchi, 2018). Continuous traits often preserve more information than the discrete characters and can hence yield higher statistical power in evolutionary studies (Table 1). Beltran and Tarwater listed “body size,” “life-history timing,” and “body mass in kilograms” as commonly measured continuous traits in evolution and ecology (Beltran and Tarwater, 2024). Similarly, Martin and Weber considered flower and leaf trait evolution, body size and generation time in eucalypt trees (Martin and Weber, 2024).

**TABLE 1:** Comparison between continuous and discrete characters.

Character types	Definition	Data types	Popular models	References
Discrete Character	Characters take a finite set of categories	Mainly qualitative (categorical data), such as binary, multistate, ordinal, nominal	Markov models: hidden Markov, structured Markov, threshold models, multinomial PGLMMs	Gerber (2019), Harvey and Rabosky (2018), Mora et al. (2025)
Continuous Character	Characters are represented on numerical scale and can have real value within a range	Primarily Quantitative data	generally modeled with Brownian motion and the OU (Ornstein–Uhlenbeck) model for the character evolution	Parins-Fukuchi (2018), Goloboff et al. (2006), Mora et al. (2025), Wright and Hopkins (2025)

Meristic characters can be presented as a distinct and “countable” trait that draws similarities from continuous and discrete characters. These characters are enumerable features of morphology that are often applied in phylogenetic studies and biological investigation, such as disciplines like ichthyology or fish biology (Lawing et al., 2008). Traditionally, meristic traits were specifically defined as those that were evolutionarily linked with body segments; however, the concept of this character is now being used more widely. These characteristics are strictly measurable, displaying discrete, integer-valued variation, for example number of fin rays, fin spines, gill filaments, or vertebrae (Waldman, 2005; Savelyev et al., 2011). Examples of commonly examined meristic traits include external anatomy, such as the number of fin rays (dorsal, anal, pectoral, pelvic and caudal) and scales, as well as internal construction, including vertebrae, pyloric caeca, branchiostegal rays and pterygiophores (Yurtseva and Zhukov, 2024). Analysis on several marine species, such as *Tilapia zillii*, *Petromyzon marinus* (sea lamprey), and *Oncorhynchus keta* (chum salmon), constantly utilized meristic feature counts to understand morphological variation, species differentiation, and identify the populations (Oladimeji and Olaosebikan, 2017; Almeida et al., 2008; Beacham, 1990). Another example includes the development of features, like teeth, which can involve a standardized hierarchy that affects how these characters advance in an ordered way, even though this culminates in a meristic count (Brocklehurst and Haridy, 2021). Fundamentally meristic features are closer to discrete traits, but their genetic basis can be quantitative, marking them as a unique type of quantitative character.

### 2.3 Topological and anatomical network analysis

Beyond geometric morphometrics, Anatomical Network Analysis (AnNA) models morphological characters as a network of interacting features. AnNA treats the anatomical parts as the elements of a graph (e.g. bones, joints, or other morphological elements) which can then be used to identify the modularity, complexity, evolvability and integration. The edges of the network are represented as their physical or functional connections.

Esteve-Altava et al. (2011) introduced the usage of the network in the field of morphology to deal with the common analyses and problems. AnNA provides a strong and resilient method for breaking down the complex connections within intricate biological structures thus facilitating the quantitative assessment of morphological homology and evolutionary paths (Strong et al., 2022). This technique allows for an independent perspective on biological entities that focuses on the interrelationships and orientations of anatomical parts instead of changes in size and shape. The focus on these topological organizations elucidates how intricate biological processes show up as morphological modularity and integration which are essential for understanding evolutionary opportunities and restrictions (Molnar et al., 2017; Asakura and Kawabe, 2022).

The method has been used in a number of studies, which have found the conservation of high-degree elements, modular partitions that correlate with morphogenetic regions, and macroevolutionary simplifying trends, which imply evolutionary constraint and possible stabilizing selection (Qin et al., 2024).

A Graph Neural Network (GNN) is a very recent class of machine learning and artificial intelligence that has gained tremendous success in processing complex relational data via handling data structured in graphical organization, including biological and anatomical networks (Xu et al., 2018; Khemani et al., 2024). Recent advances in graph-based AI, especially GNNs, offer a computational tool for combining AnNA and machine learning approaches. For the generation of task-specific embeddings for Bayesian phylogenetic inference, the GNN framework learns topological attributes of phylogenetic trees by combining node features (initialized via Dirichlet energy minimization) with message passing. This technique outperforms traditional approaches that includes the hand-designed representations (Hunt et al., 2025).

Neural networks have allowed the researchers to develop toolkits and perform robust analyses of morphological features. Adaïmé et al. (2024) developed a phylogenetic toolkit using variants of neural networks. They evaluated their model using optical superresolution micrographs of 30 *Podocarpus* species. This approach suggests that neural networks can be involved in identifying the phylogenetic history enclosed in the species morphology, thereby bridging the events of extinction and speciation.

The topological approach could be particularly useful in paleobiology, where often one is able to reconstruct only a part of the geometry of fossils. Morphometric analyses based on landmarks become challenging for fossilized material that is fragmented or incomplete, but the preserved structural relationships among the fossilized elements can still offer useful evolutionary information biologically.

Additionally, AnNA and graph-based AI techniques should be considered as co-pilots for understanding GMs and classical anatomical expertise instead of substitutes of traditional approaches.

## 3 AI techniques used in morphological and fossil data analysis

### 3.1 Machine learning in morphological studies

The growing availability of digitized fossil collections, improved imaging technologies, and large morphological datasets has encouraged researchers to explore computational approaches for studying evolutionary patterns. In recent years, machine learning (ML) has become an increasingly valuable tool in evolutionary biology and paleontological analysis. Conventionally, morphological analysis depended on qualitative statements or expert-driven landmark annotations, which have been time-consuming, demanding more labor, and challenging for scaling large datasets. ML algorithms address these constraints by providing efficient, robust, and more sensitive methods for extracting, evaluating, and comprehending morphological data (Christodoulou et al., 2020). In the context of morphological and fossil research, these methods help researchers analyze large amounts of phenotypic data more efficiently and support tasks such as fossil identification, trait analysis, and the detection of evolutionary trends (Watkins, 2024). As a result, ML approaches are gradually becoming an important complement to traditional morphological analysis. Scientists are now applying these approaches to identify and sort fossils using various measurable traits and images (Table 2). Compared to manual identification, machine learning tools can work faster and often give more consistent results. In recent studies, classifiers trained on skull and fossil images were able to group specimens into broader taxonomic categories. These results suggest that machine learning can help uncover evolutionary patterns while reducing reliance on subjective human judgment (Ye, 2025).

**TABLE 2:** Summary of the other popular models of Machine learning applied.

Methods	Application	Key results	References
Random Forest	Coral morphology and genetic lineage prediction	Outperformed traditional dimension reduction methods	Mitushasi et al. (2025)
Multimodal Machine Learning, Deep learning	Framework for morphological ML stages	Spans image segmentation and feature identification across 3 developmental stages	He et al. (2024)
Landmark-free shape analysis	Primate morphological feature extraction	Extracted family-level characteristics without landmarks	Tsutsumi et al. (2023)
Deep learning	Sex classification through the Macaque mandible	Successfully classified sex using only 139 specimens; generalized across macaque species without alignment	Morita et al. (2022)
SVMorph (Support Vector Machine)	Complex morphological character classification	Reliable classification in non-model organisms with small datasets/low resources	Teng et al. (2021)
XGBoost (gradient boosting-based method)	Comparative analysis across *Arabidopsis*species, rice, maize, and mice	Improved predictive accuracy and potential identification of genes essential for complex phenotypes across agriculture, biology, and medicine	Cheng et al. (2021)

Another important application of machine learning is the phylogenetic placement of extinct pollen morphotypes. In this research, deep learning-derived morphological features were used along with phylogenetic methods to place fossil pollen within evolutionary trees. This work demonstrated that neural network-learned traits could be transformed into evolutionary distance’s, allowing fossil specimens to be positioned in a phylogeny with Bayesian inference (Adaïmé et al., 2024).

ML techniques have also been applied to the identification of tiny fossil remains. Xu et al. used a CNN-based (Convolution Neural Network) image analysis method to classify microscopic palaeobios. The approach produced accurate results and was significantly faster than traditional manual identification, demonstrating that machine learning techniques are useful even for very small fossil samples (Xu et al., 2020).

**FIGURE 2:**
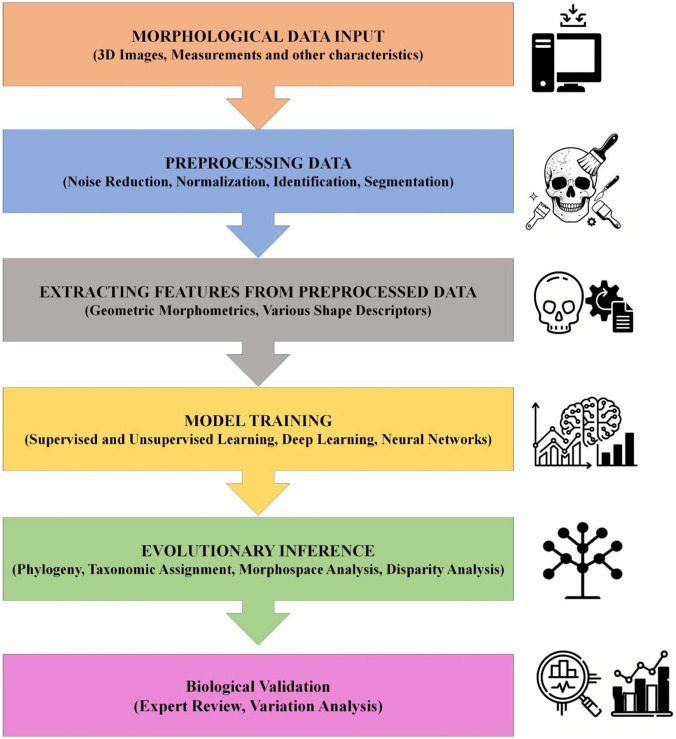
A basic walkthrough for Machine learning steps commonly employed in the Morphological studies.

Machine learning methods applied in fossil and morphological studies are commonly divided into three broad categories: supervised learning, unsupervised learning, and deep learning. Supervised learning techniques rely on datasets in which the input data are paired with known outputs, such as species identity or a particular morphological category. During training, the algorithm learns the relationship between these inputs and outputs and can later apply this knowledge to classify new samples. Common supervised algorithms used in biological studies include Support Vector Machines (SVM), Random Forest models, and Artificial Neural Networks (ANN). These approaches have been applied to a variety of tasks, including fossil classification, species identification, and the prediction of morphological traits from imaging data (Marques et al., 2025; Fischer et al., 2020). Figure 2 depicts refined steps in applying machine learning to the morphological analysis.

In cases where labeled datasets are limited, researchers often rely on unsupervised learning approaches. Unlike supervised methods, these techniques attempt to identify patterns within the data without predefined categories (Gerber, 2019). In particular, principal component analysis (PCA) is frequently used in morphometric studies because it reduces complex, high-dimensional datasets into a smaller set of variables that represent the most important patterns of variation. By projecting specimens into a morphospace, researchers can visualize how different taxa relate to each other in terms of shape or structure and identify potential evolutionary trends (Schaeffer et al., 2020).

In morphometric analysis, machine learning methods are being used to assist the investigations. Traditional morphometric studies typically involve manually identifying anatomical landmarks and recording measurements, a process that can be time-consuming and sometimes subject to observer bias (Gunz, 2019). By combining computer vision techniques with geometric morphometrics, machine learning models can automatically segment images, detect anatomical landmarks, and extract quantitative shape measurements from large image datasets (Finka et al., 2019). These advances span small datasets, landmark-free methods, and non-model organisms (Table 1).

Although machine learning offers several advantages for analyzing morphological and fossil data, some limitations remain. Fossil datasets are often relatively small or unevenly preserved, which can make it difficult to train robust models. In addition, the results produced by complex AI models may not always be easy to interpret biologically. Nevertheless, as digital fossil databases continue to grow and computational tools become more accessible, machine learning is expected to play an increasingly important role in studying morphological diversity and evolutionary history.

### 3.2 Deep learning models: a step forward to machine learning

Deep learning models, specifically convolutional neural networks (CNNs), have been broadly used for fossil image analysis. A recent study developed a CNN model to identify fossil shark teeth from image collections (Barucci et al., 2024). The model achieved high classification accuracy and demonstrated the potential for deep learning systems to assist in fossil identification tasks that were traditionally performed by domain experts.

The integration of deep learning (DL) into evolutionary science has significantly transformed the way biological history and complex biological structures are analyzed. For example, Borowiec et al., convolutional neural networks predominate in image-based identification of morphological features (496 research), and 64% of more than 800 DL studies in evolution and ecology were published since 2019 (Borowiec et al., 2022).

During the early 2020s, the focus of deep learning research in evolutionary science shifted toward reconstructing fragmented biological history. Yu and colleagues began using advanced architectures, including U-Net and DeepLab models, which were created for medical image segmentation (Table 3), to perform digital reconstruction of fossil structures. These methods allowed computers to distinguish fossilized bone structures from surrounding rock material in CT scan images, significantly reducing the time required for manual segmentation by paleontologists (Yu et al., 2022).

**TABLE 3:** Applications of deep learning in morphological studies.

Year	Study	Methods	Application	Key results
2026	Sun (2026)	ResNet34	Bird morphological disparity	Analyzed more than 10,000 bird species for quantitative disparity patterns identification
2025	Adaimé et al. (2025)	Deep learning	Pollen morphology and environment	Linked pollen traits to environmental factors across 31 fossil species
2024	He et al. (2024)	Review (Deep learning)	Data acquisition/processing framework	Deep learning dominant in image segmentation, feature recognition, morphometrics, phylogenetics
2022	Borowiec et al. (2022)	Synthesis (800+ studies)	Evolution/ecology DL trends	64% of applications published since 2019; CNNs dominant
2019	Cuthill et al. (2019)	Deep convolutional triplet networks	Butterfly Müllerian mimicry	Quantitatively validated mimicry theory among various species using 2,468 photographs

## 4 Applications of AI in morphological evolution

**FIGURE 3:**
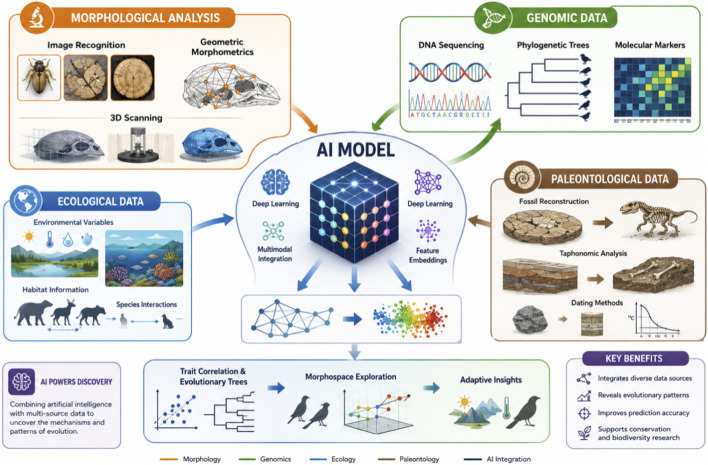
Application of AI in morphological studies and evolutionary phenomics.

Artificial intelligence acts as a computational framework for converting morphological information into representations that can be integrated with evolutionary theory. Traditionally, morphological studies relied on manual character coding and morphometric measurements. AI methods, for example, computer vision, deep learning, and generative modeling, are helping us in the automated extraction of morphological traits from images, fossils, etc., which can be used as high-dimensional data suitable for evolutionary analysis (Figure 3). These approaches facilitate scalable integration of morphology with genomic, ecological, and paleontological datasets, expanding the analytical capacity of evolutionary biology.

### 4.1 AI-driven taxonomic recognition

Taxonomic recognition is one of the most direct inferences of morphological classification and evolution. Traditionally, species can be identified with the help of experts using character coding and morphometrical analysis. Taxonomic recognition of species in evolutionary terms is traditionally time-taking, expert-driven, and subjective.

Taxonomic impediment is a worldwide shortage of taxonomic expertise, which leads to loss of data and characterization even before discovering the biodiversity. Besides conceptual, practical, and technical problems in taxonomic classification, convergent evolution, phenotypic plasticity, and cryptic species also participate in misidentifications. The adoption of AI can help close the taxonomic specialization divide by making identification easier and more affordable, offering an alternative to traditional methods. Integration of morphological classification and molecular data is still limited, which constrains traditional approaches of taxonomic identification, creating the space for integrative, data-driven, and AI-assisted taxonomy (Ebach et al., 2011; Hawksworth and Lücking, 2017; Crous et al., 2014; CBD, 2008).

Artificial intelligence can revolutionize taxonomic recognition by providing automated, highly accurate species identification and integrating data from different domains. With the digitization of biodiversity data and the establishment of global image repositories, artificial intelligence (AI), particularly deep learning–based computer vision, has emerged as a transformative tool for automated taxonomic classification.

Niu et al. (2021) reported that their success rate is 86% at the genus level and 81% at the species level when they investigated graptolite fossils. Hogeweg et al. (2024), however, developed large-scale models that took only a year to complete 65 million identifications, which include European flora and fauna. Deep learning models are the major strength of artificial intelligence, which can detect minute morphological details, provide taxonomic expertise, and process large datasets rapidly, which can help in performing complex tasks easily (Checksfield, 2019; Schermer et al., 2018). This technological advancement can not only be applied to paleontology for taxonomic recognition but can also help in entomology, herbarium specimen analysis, ecological analysis, etc.

However, AI models depend on curated and balanced training data. Shifting evolutionary studies from laboratory, museum, and field images to AI-based tools remains an active research challenge.

### 4.2 Phylogenetic and evolutionary analysis

Even if the use of genomic data is used for establishing phylogenetic and evolutionary relationships, the use of morphological traits remains vital for taxonomic classification. Molecular data cannot fully replace morphological phylogenetics. Lee et al. (2018) emphasize that morphology is an independent source and cannot be replaced by molecular information. Macleod (2002) describe morphology as the basis of phylogenetic relationships. However, the field faces significant challenges. Scotland et al. (2003) identify a limited number of characters available as obstacles in predicting homology. Assis (2009) document a decline in morphological phylogenies due to theoretical and methodological issues.

Using AI for morphological phylogenetics is increasing by using deep learning for automating trait extraction and integrating morphological data with molecular data. Recent studies demonstrate both promise and limitations. Cuthill et al. (2019) analyzed 2,468 butterfly photographs across 38 subspecies using deep convolutional networks and correlated them with gene-level phylogenies. Hunt et al. (2025) found that deep learning-derived morphological traits from insect images improved the results when both sets of data were integrated but couldn’t give results alone. Adaïmé et al. (2024) used neural networks to train existing data of 30 Podocarpus species to place 9 fossil pollen specimens. He et al. (2024) says that AI methods can help in data acquisition and processing stages, image segmentation, feature recognition, and morphometrics. However, Goswami and Clavel (2025) emphasize that while AI can help accelerate phenomic data acquisition, the need for robust evolutionary models is still there for integrating fossil data into large phylogenetic trees.

Integration of morphological data extraction with omics datasets through multimodal learning frameworks enhances the final output. Ensuring the evolutionary relevance of learned representations is essential for integrating AI into phylogenetic bioinformatics.

### 4.3 Morphospace analysis and phenotypic landscapes

Morphospace analysis and phenotypic landscapes are mathematical frameworks for studying evolutionary patterns by representing organism forms as points in multidimensional spaces. At the same time, morphospaces are mathematical representations where phenotypes are used and plotted as points, with proximity indicating morphological similarity (Mitteroecker and Gunz, 2009). However, the genotype-phenotype map is quite complex and nonlinear, complicating transitions from genotype to phenotype spaces (Pigliucci, 2010). Despite these issues, morphospace approaches can be used to identify evolutionary constraints and accessibility patterns with the help of the inclusion of developmental data (Gerber, 2025).

Morphospace analysis is used to quantify phenotypic disparity and evolutionary constraints. Conventionally, Principal Component Analysis (PCA) is used, which is helpful in low-level datasets but fails in nonlinear morphological variations. Autoencoders are being used to capture nonlinear data for dimensionality reduction, which can help us generate morphological structure representations. It can help capture high-order phenotypic relationships beyond linear projections. Generative models, including Generative Adversarial Networks (GANs), can also be useful by extending morphospace exploration by simulating hypothetical or intermediate phenotypes. Clustering methods applied to latent embeddings further facilitate the detection of morphological modules and ecological specialization.

Dinnage and Kleineberg (2025) created a generative AI model (DeepSDF) on 3D scans of around two thousand bird species’ bills and successfully predicted the trophic niche with high accuracy. Cavanagh et al. (2021) characterized morphodynamics in 2D morphospace, discovering phenotype transition landscapes.

However, the interpretation of latent variables is a tremendous task. Linking computational dimensions to identifiable anatomical features requires data analysis after the model is prepared. A strict validation is necessary to correlate AI-derived morphospaces and biologically meaningful phenotypic axes rather than algorithmic artifacts.

Recent advances in machine learning have opened new and advanced avenues for modeling complex morphological variations. Variational autoencoders (VAEs) can be used for probabilistic representations of phenotypic variations beyond linear projections (Kingma and Welling, 2019). Nonlinear dimensional reduction techniques such as Uniform Manifold Approximation and Projection (UMAP) further improve visualization of high-dimensional phenotypic data (McInnes et al., 2018). Emerging diffusion-based generative models can also help simulate hypothetical morphological forms (Ho et al., 2020).

### 4.4 Taphonomy and paleontological reconstruction

Taphonomy is essential for accurate paleontological reconstruction because it identifies and quantifies how preservation processes bias the fossil record, enabling paleontologists to distinguish original biological patterns from post-mortem artifacts. AI provides computational tools for automated fossil classification, reconstruction, and integration into evolutionary models.

Initially, Behrensmeyer and Kidwell (1985), Behrensmeyer (2026) established that taphonomic analysis sharpens the diagnosis of bias in paleontological data and contributes to further understanding. Modern advancement (Demuth et al., 2022) enabled reconstruction of original morphology despite distortion, and digital mapping systems (Chadwick et al., 2016) providing data from up to centimeter-level from bonebeds. CNN-based classifiers have been used for fossil identification from both two-dimensional images and three-dimensional micro-CT scans (Hsiang et al., 2019). These models facilitate high-throughput paleontological analysis and reduce reliance on manual morphological comparison. In addition, generative frameworks enable reconstruction of incomplete fossil specimens by predicting missing anatomical components based on learned morphological patterns.

Cifuentes-Alcobendas and Domínguez-Rodrigo (2019) achieved 95% accuracy in classifying cut marks on fleshed versus defleshed bones using computer vision. Domínguez-Rodrigo et al. (2024) attained 88% accuracy in discriminating taxon-specific African carnivore tooth marks. Jiménez-García et al. (2024) successfully applied convolutional neural networks to reconstruct taphonomic agency at Tritons Cave using transfer learning. Yu et al. (2024) reviewed over 70 paleontological AI studies since the 1980s but noted a 10–20 years gap between paleontological and mainstream AI applications. The recent increase reflects improved accessibility to AI tools rather than fundamental methodological advances.

Nevertheless, caution is required when interpreting reconstructed morphologies. Generative models may introduce plausible but biologically inaccurate structures if not constrained by anatomical knowledge. Transparent validation against expert reconstructions and independent datasets is essential to maintain paleobiological rigor.

### 4.5 Phenomic databases and data infrastructure for AI-Driven morphological analysis

Phenomic databases are integrated resources that systematically organize genotype-phenotype associations across species and experimental methods to facilitate gene discovery and functional analysis (Kahraman et al., 2005). Phenomic databases are transforming morphological evolution studies by enabling large-scale integration of phenotypic data across taxa, though adoption of standardized formats remains incomplete.

Phenomic databases act as phenomic repositories in the bioinformatics context, where we can store phenotypic traits and link them with genomic, ecological, and environmental information. Artificial intelligence systems can process them via computer vision pipelines, converting raw morphological images into feature embeddings, which can help capture complex biological data. A comprehensive analysis of genomic, environmental, and phenomic data via multimodal machine learning can help understand evolutionary dynamics in a better way. As digitized resources for biodiversity continue to expand, phenomic databases will play a critical role in supporting AI-driven evolutionary bioinformatics (Page et al., 2015; Nelson and Ellis, 2019; Heberling et al., 2021).

Various phenomic databases have been developed. PhenomicDB integrates data from different organism-specific databases (WormBase, OMIM, FlyBase, MGI), including humans and other model organisms, enabling cross-species phenotype comparison. PhenomicDB contained over 300,000 phenotypes with clustering tools for functional analysis (Groth et al., 2010). Specialized databases include PhenoM (yeast morphological phenotypes with 78,194 images) (Jin et al., 2012), PhenoDB (clinical phenotypic information for Mendelian genomics) (Hamosh et al., 2013) and PhenoScanner (human genotype-phenotype associations with 350+ million results) (Staley et al., 2016).

PhenoScanner alone has 1,241 citations, indicating strong community engagement with phenomic resources for understanding disease pathways and gene function.

The Phenoscape Knowledgebase contains over 500,000 fish phenotype annotations across 2,500+ teleost species (Mabee et al., 2012), and using ontology-based reasoning, generated a synthetic supermatrix of 639 characters for 1,051 sarcopterygian taxa with over 145,000 populated cells (Dececchi et al., 2015). MorphoBank enabled a collaboration of 25+ researchers to produce >4,500 phenomic characters supported by >10,000 media files (O'Leary and Kaufman, 2011).

Global biodiversity infrastructures such as the Global Biodiversity Information Facility (GBIF) and iNaturalist provide millions of annotated biodiversity records that can support automated species identification and phenotypic analysis using artificial intelligence.

### 4.6 Integrative computational morphology

Across taxonomic recognition, phylogenetic modeling, morphospace analysis, and paleontological reconstruction, AI functions as a unifying computational layer connecting morphology to evolutionary bioinformatics. By transforming phenotypic structure into structured, high-dimensional embeddings, AI enables scalable integration of morphological, ecological, and molecular data.

Future developments are likely to include multimodal learning systems that combine morphology with genomics, transformer-based phenotypic representation models, self-supervised learning on large biodiversity image repositories, and explainable AI frameworks tailored to evolutionary datasets. Ensuring interpretability, reproducibility, and standardized data infrastructures will be central to advancing AI-driven morphological evolution research.

### 4.7 Machine learning in fossil species distribution models (SDMs) and ecological niche modeling

Species distribution models (SDMs) are models which predict a species’ distribution and range and are commonly used to provide a quantitative baseline for conservation. Most SDM frameworks and approaches, however, have been fairly well established for species with broad continental distributions and relatively large numbers of occurrence records (Rios et al., 2024). These models incorporate statistical learning techniques that numerically capture the relationship between the environmental factors and biotic responses and then use this numerical relationship to predict the suitability or probability of occurrence of the habitat across space (Franklin, 2013). SDMs and other similar ecological niche models are now important tools in reconstructing past species ranges, climates and communities. They are an efficient tool for paleoecology and can be used in combination with fossils, genetics and climate models to better understand the response of species and ecosystems to environmental change in the past. SDMs generate quantitative, high-resolution maps of past distribution beyond sparse fossil localities.

They are broadly employed for the Reconstruction of Pleistocene glacial refugia, range shifts, and constraints on current patterns of diversity and have been applied since the Holocene up to 400+ Ma (Svenning et al., 2011; Blois et al., 2025). SDMs can aid in the identification of true absence of fossils vs. sampling bias; they can also expose sampling bias artifacts in apparent diversity patterns (such as the apparent absence of dinosaurs before the K–Pg extinction) (Chiarenza et al., 2019).

Currently, machine learning is a mainstay of SDM and ecological niche modeling (ENM), and a number of studies further build on and apply these concepts to fossil and paleoecological and deep-time biogeographic studies. According to the systematic review conducted by Miyaji et al. (2025), main machine learning (ML) models used widely in SDM are Random Forest and Maximum Entropy (MaxEnt). These tools are also modified for the paleoecological and biogeographic reconstruction when data are sparse and biased by using climate-driven hindcasts, co-occurrence and recommender models, trait-based classifiers, and taxonomic refinement with ML tools.

Random Forest (RF) classifier was trained on modern trophic structure links that were hindcast over 120,000 years to project variation in mammal trophic guild assemblages and compare them with fossils (González-Trujillo et al., 2025). Linchamps et al. (2023) trained another RF model that was claimed to outperform other ML models. It was used to predict climate variables explicitly designed to be transferable to paleo-faunal communities despite taxonomic uncertainty. Romero et al. (2020) developed three convolutional neural network (CNN) classification models which they trained on pollen of 16 genera of the legume tribe Amherstieae. This fossil identification model supports the paleobiogeographic hypothesis of Amherstieae being originated in Paleocene Africa and dispersed to South America during the Paleocene-Eocene Thermal Maximum.

Beyond conventional ML-driven SDMs, recent advancements have incorporated phylogenetic data directly into ecological niche modeling architecture. Mondanaro et al. (2023) introduced a new algorithm ENphylo, which is a readily available R package, *RRdtn*. It is a phylogenetically informed algorithm structured to model the distribution of rare species by incorporating evolutionary relationships into ecological niche predictions. ENphylo has proved to be more accurate than Environmental Niche Factor Analysis (ENFA) and other bivariate models like MaxEnt, Generalized Linear Models (GLMs) and RF classifier. ENphylo has higher predictive accuracy for sparse occurrence data sets, which is especially important in fossil or paleontological biogeography where records of species are intrinsically incomplete and need imputation.

Similarly, as far as macroevolutionary climate models are concerned, they have grown in popularity for studying the impact of broad-scale climate changes on evolutionary trends and dispersal of a species. For instance, Timmermann et al. (2024) investigated the impact of past climate change on human evolution, combining paleoclimate modeling with fossil records and evolutionary modeling techniques, thus revealing the increasing use of computational methods for reconstructing evolutionary responses to environmental change. The study has also discussed the diversification and expansion of the hominin climate niche towards colder climates as an important representative for climate related adaptations in various *Homo* species.

## 5 Major gaps in current machine and deep learning approaches

Despite the advancement in ML/DL techniques and applications in morphological evolution, it faces a notable limitation that restricts the adoption widely.

- Data Acquisition and Processing Gaps: Computational morphological studies highly rely on the datasets, which are not yet fulfilled. The ability to obtain data is quickly outpacing the ability to analyze it with robust, realistic evolutionary models, bringing a new set of challenges. The lack of automation tools for data extraction leads to the underrepresentation of the discipline.
- Performance Gaps and Phylogenetic Signal: While being informative, deep learning-derived morphological characters underperformed when used in isolation compared to molecular analyses. Such performance leads to weaker phylogenetic signals. Features derived from DL can have phylogenetic signals but are difficult to assign to biologically meaningful characteristics, making evolutionary interpretation difficult (He et al., 2024).
- Explainability and Interpretability Gaps: Although highly accurate classification is desirable, it does not help to advance our understanding of evolutionary processes by itself. Scientific value of a model with 95% accuracy in taxon recognition (Bowman et al., 2026) can only be gained by the ability to query the model and obtain the information as to which morphological features are responsible for the recognition of the taxon and whether the features correspond to biologically significant characters. A number of XAI (Explainable AI) methods are now being adopted for morphological studies. Gradient-weighted class activation mapping (Grad-CAM) generates saliency maps that show where the CNN focuses when making its classification, which can permit the researchers to check whether the CNN is paying attention to morphologically significant anatomy or to preservational artifacts like matrix texture (Fayyaz et al., 2025). SHAP (SHapley Additive exPlanations) values can help in breaking down the contribution of each individual morphometric measurement or landmark coordinate to the output of a model, connecting these predictions of deep learning and the interpretable character-state logic of comparative anatomists (Ponce-Bobadilla et al., 2024). The adoption of XAI in the context of paleontological AI pipelines is more than just a technical advancement, it's a necessity for maintaining the integrity and trustworthiness of scientific research. In absence of interpretability, high accuracy models remain black boxes and their outputs cannot be meaningfully peer-reviewed (Korfmann et al., 2023).
- Methodological and Technical Gaps: Dou et al. (2023) argued that small data challenges are technically more problematic in ML/DL studies but the fossil record poses an especially severe version of this problem. Paleontological datasets are not merely small as they are fragmentary, taxonomically uneven and shaped by taphonomic filters that differ across geological periods and depositional environments. There is a need for methods that are tailored to sparse data with missing values. Few-shot learning (He et al., 2024) is particularly promising where models are able to learn to generalize from a few labeled examples per class and the meta-learning technique model-agnostic meta-learning (MAML) might enable a well-sampled set of rare taxa to be quickly adapted when a fossil genus with less than ten specimens is described (Finn et al., 2017). Siamese networks and prototypical networks are able to learn morphological similarity metrics that are robust to the extreme class imbalance common in paleo collections (Yu et al., 2024). These are strategies that are notably more advanced than those of transfer learning already mentioned in the taphonomy section and are a qualitatively different way of dealing with the data scarcity that is intrinsic to the fossil record as opposed to being a byproduct of limited digitization.
- Gaps in Model Developments: Multimodal learning in morphological studies is only beginning to be explored, which needs much attention to counter the issues, such as automation problems and the availability of big data for phylogenetic analyses. A critical underexplored gap is the integration of ML with biogeographic and ecological data for reconstructing the past distributions of extinct or extant taxa. Big data models and datasets in genomics and phylogenetics strain computing resources and training efficiency is a major bottleneck in the development of particular models (Sapoval et al., 2022). The integration of molecular and morphological data is still one of the big challenges in evolutionary biology because the two types of data have widely different structures, scales, dimensions and incompleteness. Molecular data are frequently high-dimensional and based on sequence, while morphological data are relatively few and sparse and heavily determined by preservational bias in fossils (Guillerme and Cooper, 2016). In addition, often extinct organisms have no molecular information at all, so that phylogenetic inference is mainly based on morphology. Another challenge is the differences in evolutionary rates between molecular and morphological data sets and the conflicting signals found in the trees (Holvast et al., 2024). There are some recent multimodal ML models that offer potential solutions to integrate these different data types but standardized pipelines for integrating them are limited.

## 6 AI as co-pilot: a complementary partner

In this review, AI and ML technologies have been described as tools that speed up and scale morphological analysis. This framing must be clear that it is not meant to replace classical paleontological skills but rather complement them. The models of AI rely heavily on the data generated by human experts over many years such as fossil preparation, anatomical description, scoring of characters, building of a phylogenetic matrix are all inputs which an AI model would not be able to operate without. In evolutionary morphology, image capture, segmentation, feature recognition, morphometrics and the inference of phylogeny are already major uses of AI methods, such as classical ML and deep learning as well as newer large-scale or multimodal models, to allow big data evolutionary phenomics (He et al., 2024). The multi-agent LLM platforms, which work alongside human experts and specialized computational tools, are yet to be tested and monitored and remain experimental. AI has the potential to change the way evolutionary biology works, from data-limited to idea-limited science and to pose important queries about transparency, skills and equitable access to the benefits of AI, as the use of these tools becomes more sophisticated.
